# Sources of Artifacts in SLODR Detection

**DOI:** 10.11621/pir.2021.0107

**Published:** 2021-03-31

**Authors:** Aleksei A. Korneev, Anatoly N. Krichevets, Konstantin V. Sugonyaev, Dmitriy V. Ushakov, Alexander G. Vinogradov, Aram A. Fomichev

**Affiliations:** a Lomonosov Moscow State University, Moscow, Russia; b Institute of Psychology of Russian Academy of Sciences, Moscow, Russia; c Taras Shevchenko National University of Kyiv, Kyiv, Ukraine; d TalentCode Consulting Company, Moscow, Russia

**Keywords:** intelligence, Spearman’s law of diminishing returns, mathematical modeling, structural modelling, structure of intelligence

## Abstract

**Background:**

Spearman’s law of diminishing returns (SLODR) states that intercorrelations between scores on tests of intellectual abilities were higher when the data set was comprised of subjects with lower intellectual abilities and vice versa. After almost a hundred years of research, this trend has only been detected on average.

**Objective:**

To determine whether the very different results were obtained due to variations in scaling and the selection of subjects.

**Design:**

We used three methods for SLODR detection based on moderated factor analysis (MFCA) to test real data and three sets of simulated data. Of the latter group, the first one simulated a real SLODR effect. The second one simulated the case of a different density of tasks of varying difficulty; it did not have a real SLODR effect. The third one simulated a skewed selection of respondents with different abilities and also did not have a real SLODR effect. We selected the simulation parameters so that the correlation matrix of the simulated data was similar to the matrix created from the real data, and all distributions had similar skewness parameters (about –0.3).

**Results:**

The results of MFCA are contradictory and we cannot clearly distinguish by this method the dataset with real SLODR from datasets with similar correlation structure and skewness, but without a real SLODR effect. The results allow us to conclude that when effects like SLODR are very subtle and can be identified only with a large sample, then features of the psychometric scale become very important, because small variations of scale metrics may lead either to masking of real SLODR or to false identification of SLODR.

## Introduction

I n 1927, British psychologist Charles Spearman (Spearman, 1927; Detterman & Daniel, 1989) formulated the hypothesis that, when measuring intellectual ability, one finds higher subtest correlations in the lower region of general factor (*g*) distribution and, vice versa, lower subtest correlations in the higher region of the *g* distribution (the so-called Spearman’s Law of Diminishing Returns, SLODR). Testing and discussion of this hypothesis continued, and has even increased over the past three decades. Many studies involving widely varying types of respondents, tests, and data-processing methods have been published over that period. The results were discordant.

Although the tendency for a decrease of intercorrelations between the subtests when the *g* factor is growing has been verified in a meta-analysis, only a little more than half of the studies reviewed directly confirmed Spearman’s hypothesis (Blum & Holling, 2017). There is some criticism of this meta-analysis in a study by Hartung and colleagues (Hartung, Doebler, Schroeders, & Wilhelm, 2018). The first point of the criticism is that the meta-analysis did not include recent studies with new methods for examining the SLODR hypothesis. Indeed, the question of which statistical methods are appropriate to investigate the structure of intelligence is very important. It seems that the SLODR effect in general is not strong, and the ways to detect it statistically may not be so trivial as was originally supposed. To examine the differentiation and dedifferentiation of intelligence along the age dimension and/or growth of ability, current studies use the following methods: confirmatory factor analysis, moderated factor analysis (Molenaar, Dolan, Wicherts, & van der Maas, 2010), multi-group confirmatory factor analysis (Reynolds & Keith, 2007), factor mixture modeling (Reynolds Keith, & Beretvas, 2010), and local structural equation models (Hildebrandt, Lüdtke, Robitzsch, Sommer, & Wilhelm, 2016) (for a brief review, see the introduction to Hartung et al., 2018). We believe that each of these procedures is worth a separate discussion in the context of SLODR detection, but in our study we will focus on moderated confirmatory factor analysis (MCFA). The goal of our study is to investigate the capabilities of MCFA to detect SLODR under different conditions.

As the SLODR effect is not strong, it can be statistically confirmed only when large data samples are used. We have seen such very large samples in SLODR studies (for instance, Arden & Plomin, 2007; Breit, Brunner, & Preckel, 2020; Dombrowski, Canivez, & Watkins, 2018; Hartmann & Reuter, 2006; Hartung et al., 2018; McGill, 2015). Using large samples not only allows the researcher to obtain more statistically reliable results, but it also enhances the probability of the appearance of artifacts (Korneev, Krichevets, & Ushakov, 2019). In particular, artifacts may be generated by the scale characteristics and skewed distributions of scores.

In a field close to SLODR study — the study of gene–environment interactions — artifacts have become the object of deep reflection and special investigations. Analysis so far indicates that some of the results may be explained equally by either real interactions or subtle features of the distributions. For instance, A. Murray and colleagues conclude that “Estimates of gene–environment interactions (G×E) in behavioral genetic models depend on how a phenotype is scaled. Inappropriately scaled phenotypes result in biased estimates of G×E and can even suggest G×E sometimes in the direction opposite to its true direction” (Murray, Molenaar, Johnson, & Krueger, 2016, p. 552). The authors also point out two reasons for the violation of normal distribution of phenotypic characteristics, which then often lead to ambiguity in the measuring scales. These are the irregularity of the distribution of tasks according to their difficulty, and selection of respondents according to their abilities. “The problem of dependency of G×E on phenotype scaling has been known since the time of R.A. Fisher who noted that G×E interaction could be manipulated by re-scaling the variable involved” (Murray et al., 2016, p.553). Re-scaling here refers to a non-linear but monotonic scale transformation, which is permissible for ordinal scales of measurement as defined by S. Stevens (Stevens, 2017), but not for interval scales. However, only the latter can be used in the great majority of complex mathematical measurements (for instance, in modeling by linear structural equations and in linear regression). Nevertheless, a great number of cases where those methods are employed do not contain any serious arguments in favor of interval scales.

Some researchers who understand the importance of this problem opt for using measurement methods based on Item Response Theory (IRT) (Breit, Brunner, & Preckel, 2020; Embretson & McCollam, 2000, Tucker-Drob, 2009). For instance, I. Schwabe argues that the IRT approach generally provides greater reliability than the summation of scores (Schwabe, 2016). Another approach is using item scores in factor analysis directly (Molenaar, Ko, Rózsa, & Mészáros., 2017). In our work, we do not discuss in detail the applications of IRT in the context of the SLODR effect, but we think it is an important direction of investigation.

In SLODR studies we see, first of all, interest in the effect of distribution skewness on SLODR detection. Murray, Dixon, and Johnson (2013) point out that subtest distribution skewness may result in SLODR detection when the effect is actually absent. The sources of the skewness are the same as in Murray et al. (2016): the selection of respondents in the data set, which is defined by external factors, and the difference in the number of easy and difficult tasks presented in a subtest (with floor and ceiling effects as special cases).

As we show in this paper, these are the very different sources of the very different aspects of SLODR detection, and the problem is not in the skewness itself, but in the sources of deformation of the distribution.

Now, let us consider the SLODR detection methods in more detail.

The most interesting case for testing the SLODR hypothesis is when there is one data set with a continuous spectrum of respondents’ test results. In this case the hypothesis is that there will be a weakening of the interdependence of the subtests that may be expressed in intercorrelations that weaken along with the growth of the respondent’s intellectual ability. But if there is just one data set, it is difficult to explain what could be considered subtest correlation in different regions of the single set.

An earlier method of studying SLODR (which is now called “traditional”) employs principal component analysis of the subtests. The first component (obtained with no rotation) is interpreted as the general intellect factor *g*. The SLODR may be expressed in several modes:

1. Denser distribution of respondents’ *g*-factor scores at higher levels of g. If the subtest results have the standard normal distribution (or at least equal variances), then the *g-*factor scores have negative skewed distribution (Molenaar et al., 2010, Murray et al., 2013).
2. After dividing samples into two halves according to the median of *g* factor scores, factor analysis is carried out separately for each group (see, for instance, Reynolds & Keith, 2007). Finding less eigenvalue of the first factor (less variance) and less average value of factor loadings by subtests on this factor for a high *g*-value group is an SLODR marker in this case.
3. The average subtest intercorrelation (as obtained in item 2) may be compared for the two groups. The high intercorrelation average for the low *g*-value group also may be considered as evidence in favor of Spearman’s law (Hartmann & Reuter, 2006; Sugonyaev & Radchenko, 2018). The variants of the method use some other variable to divide the whole sample, and then exclude it from the analyzed set of data.

In all three cases, the supposition that the values of all the mentioned indicators are different on subsamples due to the presence of the SLODR effect, is based upon the presupposition that all subtest distributions are symmetrical; otherwise, differences in the SLODR indicators in the high and low groups might be generated by distribution skewness of any origin (Murray et al., 2013). As was shown in the 2019 study by Korneev, Krichevets, and Ushakov of “traditional” SLODR detection, skewness from different sources will tend to produce different results, expressed in different combinations of the properties outlined in the three modes.

**Figure 1.**
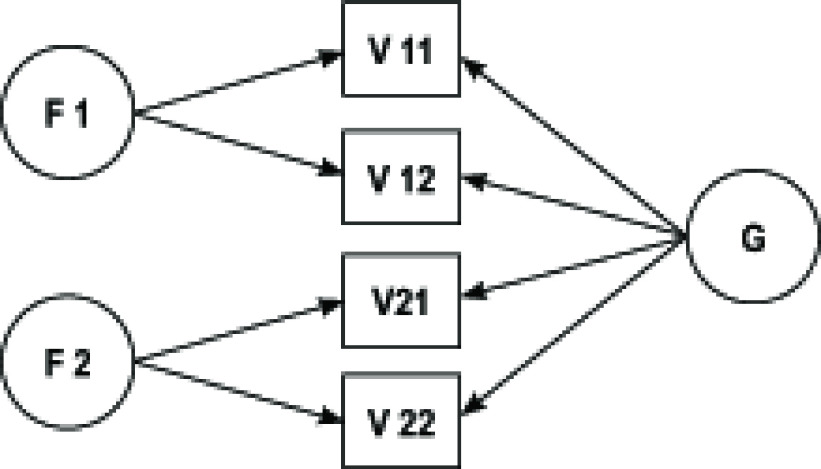
The tested bi-factor model.

*Note. V11–V22 are results of (sub)tests (real and simulated); f1 and f2 are factors of special abilities; g is the general factor.*

The so-called modern methods employ structural equation modelling. There are two types of models in use: (a) second-order or higher-order models (in which *g* is loaded only by the factors of special abilities, which in turn are loaded by the subtests); and (b) bi-fact or or nested models, in which *g* is directly loaded by the subtests, and then each of them loads its factor of special abilities. The advantages and disadvantages of the two types of models are discussed by Gignac (2016) and Molenaar (2016), but this question is beyond the scope of this article. We consider here only the second type (*Figure 1*).

In such models, Spearman’s law may be expressed, first of all, in the factor loadings being lower for an increasing level of the general factor (*g*), i.e., in a decrease of factor loadings of the subtests on the factors of special abilities, and/or on the *g* factor along the growth of the *g*-factor score. As *g* increases, residuals (on the level of subtests) may be expected to increase. The negative skewness of *g*-factor score distribution may be connected with all those phenomena (Molenaar et al., 2010; Murray, Dixon, & Johnson, 2013). The modern method of SLODR detection, moderated factor analysis, allows us to uncover such phenomena (Bauer, 2017; Bauer & Hussong, 2009). It uses the moderation of structural model parameters, i.e., the linear dependence of model parameters (factor loadings or/and residuals and so on), on factor *g* or other moderators introduced. The task is to estimate coefficients of a parameter’s linear dependence on the moderator.

In many current studies, researchers are using a hybrid methodology: The analysis of real data is compared with the results achieved by the same method applied to simulated data, with similar parameters and clear structure. When structural modeling for simulated sampling is performed to assess the same parameters that were used in sample generation, this operation does not always lead to values of the parameters close to those used in the simulations (Molenaar, Dolan, Wicherts, & van der Maas, 2010). That result shows the limits to the sensitivity of the method, so this methodology is a good supplement to the real data analysis. Moving in parallel with Molenaar et al. (2017), we constructed simulations of data with different sources of skewness and then compared the results of the application of the above-mentioned methods to the simulations and natural data.

Our research question can be formulated as follows:

Can the moderation by the *g* factor of factor loadings or residuals in moderated confirmatory factor analysis help to distinguish different sources of skewness of data? And, as a result, is it possible to distinguish a real SLODR effect from similar data patterns with other sources? Beside simulation of “true” SLODR, we simulate the situation of skewed distribution of respondents’ ability and the situation of skewed distribution of item difficulty. We analyze the moderation coefficients in MCFA of raw simulated and real data sets and these sets after normalization and try to check:

1. Whether MCFA can differentiate the sources of skewness in the simulated data sets and real data;
2. If we have skewed the real data of intelligence testing, can we differentiate the contribution of different sources of the skewness to the SLODR effect using MCFA?

## Methods

### Data

#### Real Data

The test data of 11,335 military school recruits were used in this study. The test battery of intellectual abilities, which was specially designed for the candidate selection, consisted of 10 subtests, each of which included 30 tasks. A more detailed description appeared in studies by Korneev, Krichevets, & Ushakov (2019) and Sugonyaev & Radchenko (2018). We chose four subtests with a simple correlation structure for our comparative investigations, in order to make it possible to obtain similar correlational structures in the simulations. These subtests were: (a) analogies (An); (b) syllogisms (Syl); (c) memorization of shapes (SM); and (d) verbal memory (VM). Every task required the choice of one answer out of five suggested ones. The respondents’ scores were evaluated in two ways: by the classical procedure (number of correct answers), and by the two-parameter IRT method.

The process of IRT analysis was as follows:

First, we estimated the difficulty and the discriminative ability of every task, and measured the ability score for each respondent on every scale. We used the two-parameter logistical model from the mirt R package (Chalmers, 2012; Lord & Novick, 1968).

The next step was to exclude guessers from the data. We assessed the probability of a right answer for each respondent on each item, and if the probability was lower than 0.2 (that is, the probability that the person will guess the right answer among five proposed ones) and the respondent gave the correct answer, that respondent was marked as a potential guesser on this item. Those respondents who were marked as guessers more than 10 times within one scale, were excluded from the analysis. In fact, the probability of guessing is often greater than 0.2, because some of the proposed answers could be easily rejected, so it is not surprising that our algorithm found as many as 164 guessers (1.44% of the sample), so the final sample size was 11,171. Next, we repeated the estimation of task parameters and the ability of each respondent with the updated model on the clear sample.

#### Simulated Data

In addition to the sample of real data, three sets of simulated data were produced. The random sample generation and data processing were based on SPSS version 22. The scripts for generating the data are available in Appendix S1 at http://mathpsy.com/slodr. We tried to select the parameters of simulation so that the correlation matrices of simulated data were similar to those of the natural data, and so that the variables had similar coefficients of skewness simultaneously.

##### Simulation of the selection of respondents according to the external criterion (case selection)

During the first step, a sample of 17,000 cases from a four-dimensional normal distribution with definite correlation structure was selected. Intra-group correlations were R_12_ = .71 and R_34_ = .66; four intergroup correlations (R_13_, R_14_, R_23_, R_24_) were approximately equal to .57. The first factor score (using principal component analysis) for each “respondent” was calculated. Then, the respondents with factor scores of under 0.3 (12,218 cases) were selected. The distributions of variables we obtained had negative skewness.

##### Simulation of the skewed distribution of tasks according to their difficulty (different task density)

Four standard normal variables (the correlations between them were similar to the correlations of our real data) were transformed by the following formulas: for OLD_i_ < 0, NEW_i_ = -(-OLD_i_)^1.08^; for OLD_i_ > 0, NEW_i_ = (OLD_i_)^.^93 The transformation caused the negative skewed distribution (the left semi-axis was stretched, and the right one was compressed). That distribution corresponded to a higher density of easy tasks than of difficult ones. (In reality, the modelling of different task density requires an additional supposition about the interdependency of respondents’ answers to tasks with equal difficulty. Our stretching/compression corresponds to a strong correlation.) The size of the obtained sample was 11,338.

##### Simulation of the “true SLODR.”

An additional sample was obtained using a model with decreasing factor loadings and growing residuals along increasing *g*-factor scores (the script is available in the supplementary materials, Appendix S2). The size of the simulated sample was 10,000.

We also normalized the measured variables of real data and simulated variables for the “case selection” set. The value of normalized variable V for the given case X, with a range R among all values of V from our set containing N cases, was calculated as ϕ^-1^((R-0.5)/N), where ϕ(x) is the distribution function for standard normal distribution.

### Construction and Assessment of the Model

We constructed a simple bi-factor model (see *Fig. 1*) with four indicators (real or simulated results of four subtests), two factors of special abilities (f1 and f2), and one general factor. The variance of latent factors was fixed to 1 in the models, to allow scaling and identification of latent variables. Then we estimated the same model using seven data sets: (a) raw real data; (b) normalized real data; (c) IRT real data; (d–f)) three simulations; and (g) one normalization of the simulated sample.

Then for every data set we performed principal component analysis and used the first factor scores as moderators. The following types of moderation were used: (a) the moderation of factor loadings from indicators on special abilities; (b) the moderation of residuals from the indicators; and (c) the moderation of both factor loadings and residuals from the same indicators.

In order to compare the baseline model without moderation with the moderated models (they can be considered as nested models), we used the Bayesian Information Criterion (BIC) (Bollen, Harden, Ray, & Zavisca, 2014). This criterion is relative and does not have a standard scale, but a lower BIC is a sign of a better fit. The difference in fit between two nested models can be considered significant if it is greater than 10 (Raftery, 1995).

We assessed our models in Mplus 8.3, using maximum likelihood estimation with robust standard errors (MLR), and used R version 3.6.0 (R Core Team, 2016) with the MplusAutomation package (Hallquist & Wiley, 2018) for automated processing of the model and summarizing of the results.

## Results

### Correlations and Skewness in the Real Data

The matrix of correlations is shown below in *Table 1*. The bi-factor model, with factor *g* loaded by all variables and two factors of special abilities loaded by pairs of variables, corresponds to such a structure.

**Table 1:** The matrix of correlations of the four variables included in the analysis

Scale	An	Syl	VS	VM
An	1	.536	.349	.323
Syl	.536	1	.289	.311
VS	.349	.289	1	.464
VM	.323	.311	.464	1

The coefficient of skewness is -.309 for the variable of our real An, and -.022 after normalization. It is -.232 and -.002 for Syl; -.302 and -.032 for VS; and -.361 and -.066 for VM, respectively.

### Skewness and Correlations in the Simulated Data

#### Simulation of Case Selection

Within this sample, the relatively high values of the test indicators were represented by a greater number of “respondents.” The coefficients of skewness of the four “truncated” variables fluctuated around the mean value of -.305 (SD = .023). Correlations inside the subgroups were: R_12_ = .55 and R_34_ = .48; the mean intergroup correlation (R_13_, R_23_, R_24_, R_34_) was .33 (SD = .064). For the normalized data, R_12_ = .53 and R_34_ = .46; the mean intergroup correlation (R_13_, R_23_, R_24_, R_34_) was equal to .39 (SD = .054).

#### Simulation of Different Task Density

Within this set of the data, the coefficients of skewness fluctuated around -.298 (SD = .015). The intragroup correlations were: R_12_ = .53 and R_34_ = .48; the intergroup ones were equal on average to .32 (SD = .048).

#### Simulation of the “True SLODR”

As a result, the intragroup correlations were .54 and .54, and the intergroup ones were .33 (SD = .014). The variables were standardized (they did not need any normalization since the sampling distribution differed little from the normal one; the absolute value of skewness did not exceed .03).

### Testing of the Model on Real and Simulated Data

The results are presented in *Table 2*.

The results of MCFA showed that the coefficients of moderation of factor loadings or residuals or both are often significant, but the patterns of moderation vary in different simulations. Before we discuss the specifics of these patterns, let us recall that the simulations of *different task density* and *case selection* as distinct from “true” SLODR have been constructed without the SLODR effect. Starting from a bivariate normal distribution, the first of these is obtained by simple scale deformation; the second is obtained by case selection with skew in favor of more productive “persons”.

**Table 2:** The results of moderation

Variable moderated	Δ BIC	When moderated separately		When moderated together	
		Factor Loading	Residual	Factor Loading	Residual
Data set 1. “True” SLODR (non–skewed distribution) (Chi–Sq(1) = 1.041, RMSEA 0.002 [0.000, 0.027], CFI 1.000)					
V11	–9; 0; –43	–0.031* (0.008)	0.028* (0.009)	–0.061* (0.009)	0.070* (0.011)
V12	3; –10; –53	–0.018* (.008)	0.038* (0.009)	–0.046* (0.009)	0.069* (0.011)
V21	–2 –3; –33	–0.024* (.008)	0.032* (0.009)	–0.053* (0.009)	0.067* (0.011)
V22	–5; –9; –48	–0.029* (.008)	0.039* (0.009)	–0.058* (0.009)	0.075* (0.01)
Data set 2. Simulation of different task density (skewed, not normalized) (Chi–Sq(1) = 0.710, RMSEA 0.002 [0.000, 0.023], CFI 1.000)					
V11	–12; –167; –169	–0.033* (.008)	–0.108* (.008)	0.028* (0.009)	–0.127* (0.010)
V12	–11; –138; –30	–0.033* (.008)	–0.100* (.008)	0.021 (0.015)	–0.108* (0.014)
V21	4; –123; –136	–0.018* (.008)	–0.100* (.009)	0.042* (0.010)	–0.128* (0.011)
V22	–28; –48; –38	–0.046* (.008)	–0.112* (.009)	0.001 (0.009)	–0.112* (0.010)
Data set 3. Simulation of case selection (skewed, not normalized) (Chi–Sq(1) = 0.841, RMSEA 0.002 [0.000, 0.026], CFI 1.000)					
V11	–63; 4; –57	–0.063* (0.008)	–0.021* (0.009	) –0.021* (0.009)	0.020* (0.010)
V12	–124; –17; –114	–0.086* (0.008)	–0.049* (0.01)	–0.049* (0.010)	0.003 (0.011)
V21	–67; 4; –59	–0.065* (0.008)	–0.02* (0.009)	–0.02* (0.009)	0.020* (0.010)
V22	–105; 5; –108	–0.08* (0.008)	–0.018* (0.009	) –0.018* (0.009)	0.036* (0.010)
Data set 4. Simulation of case selection (normalized) (Chi–Sq(1) = 0.982, RMSEA 0.003 [0.000, 0.027], CFI 1.000)					
V11	–35; –15; –11	–0.051* (0.009)	0.044* (0.009)	–0.085* (0.009)	0.094* (0.010)
V12	–47; –16; –135	–0.059* (0.009)	0.046* (0.009)	–0.094* (0.009)	0.101* (0.010)
V21	–33; –20; –115	–0.05* (0.008)	0.049* (0.009)	–0.084* (0.009)	0.096* (0.010)
V22	–54; –25; –172	–0.062* (0.009)	0.052* (0.009)	–0.103* (0.009)	0.113* (0.010)
Data set 5. Real data (negative skewed, not normalized) (Chi–Sq(1) = 41.261, RMSEA 0.060 [0.045, 0.076], CFI 0.995)					
An	–54; –175; –166	–0.054 (0.006)	–0.134* (0.011	) –0.005 (0.008)	–0.13* (0.014)
Sil	–24; –45; –41	–0.041* (0.007)	–0.067* (0.008	) –0.020 (0.009)	–0.054* (0.011)
SM	–30; –228; –219	–0.046* (0.006)	–0.186* (0.011	) 0.007 (0.008)	–0.191* (0.015)
VM	–90; –290; –279	–0.073* (0.007)	–0.188* (0.01)	–0.009 (0.01)	–0.181* (0.014)
Data set 6. Real data (normalized) (Chi–Sq (1) = 41.962, RMSEA 0.060 [0.045, 0.076], CFI 0.995)					
An	7; 9; 10	–0.012 (0.007)	0.007 (0.008)	–0.023* (0.009)	0.024* (0.011)
Sil	–5; –4; –20	–0.026* (0.007)	0.019* (0.008)	–0.046* (0.009)	0.047* (0.01)
SM	2; 9; 10	–0.019* (0.007)	–0.05 (0.010)	–0.023* (0.008)	0.010 (0.012)
VM	–25; 0; –16	–0.042* (0.007)	–0.029* (0.009	) –0.042* (0.009)	–0.001 (0.012)
Data set 7. Real data (IRT score) (Chi–Sq(1) = 46.663, RMSEA 0.066 [0.051, 0.083], CFI 0.993)					
An	–62; –126;–117	–0.064* (0.008)	–0.111* (0.009)	–0.007 (0.013)	–0.105* (0.015)
Sil	–155; –367; –375	–0.100* (0.007)	–0.202* (0.007)	–0.037* (0.009)	–0.179* (0.010)
SM	4; –8; 1	–0.017* (0.007)	–0.046* (0.011)	–0.001 (0.009)	–0.045* (0.014)
VM	5; 10; 4	–0.015 (0.008)	–0.004 (0.010)	–0.019 (0.010)	0.010 (0.013)

Note. * significant coefficients (p-value < 0.05). In column ΔBIC the differences between correspondent model BIC and ‘baseline’ model BIC (a negative number corresponds to a better fit with the moderated model) are presented for three variants of moderation. After the regression coefficient, the estimated standard error appears in brackets. The indicators of model fit are placed after the data set number and characterize the baseline models. The next four columns contain regression coefficients (RC) for four moderations of (a) factor loadings being moderated alone; (b) RC for residual being moderated alone; (c) and (d) RCs for factor loading and residual respectively, moderated simultaneously. The four strings contain information for the four variables.

Note that the normalization of the first one returns the distribution to the original symmetrical state, so we do not include it in our comparison. The second distribution normalization leads to a more interesting distribution symmetrization, which is created by deformation of the original scale. The result of the deformation is a product of the neutralization of two opposite asymmetrizations, neither of which can actually produce the SLODR effect.

*Table 2* contains the coefficients of moderation of different latent variables, with the first factor scores as moderators. These scores were obtained by principal component analysis of the four measured variables and have a strong correlation with latent *g*.

*Data set 1* (“true” SLODR) demonstrates the expected decreasing of factor loadings and increasing of residuals, all in accordance with Spearman’s hypothesis. In this case, the moderation effect increases when the parameters are moderated together. These data give an example of what may be seen as a “good SLODR effect.”

In *data set 2* (“different task density”), we see stronger moderation coefficients in factor loadings moderated alone than in the “good SLODR case.” As expected, residuals decrease (in a direction opposite to that of the SLODR), because the left side of the scale was stretched and the right one was compressed, which implies corresponding changes in residuals. When these parameters are moderated together, the factor loadings lose the decreasing tendency and even change it to increasing, and the residuals decrease more strongly than when being moderated alone. A similar effect may cause the inconsistent heteroscedasticity of residual variances (unexplained in Molenaar, 2011), although all other parameters show an effect consistent with Spearman’s hypothesis.

In *data set 3* (“case selection”), the picture is contradictory. It may be proposed a priori that neither factor loadings nor residuals have to show any significant moderation coefficient, but the model shows the strongest decreasing of factor loadings among all our sets of data, and not very strong *decreasing* of residuals in the case of separate analysis of these parameters. If moderation is estimated for both parameters together, then the decrease of the factor loadings becomes weaker, and residuals become about constant (which corresponds to the real residuals of our simulation).

In *data set 4,* which was derived from data set 3 by the normalization procedure described above, we have a result very similar to the “true SLODR” set in all moderated parameters. This most interesting case shows the importance of the skewness source. The negative skewness of data set 2 is accompanied by residual decreasing in the positive part of the *g* scale, and also the negative skewness of data set 3 is accompanied by equal residuals for the entire *g* scale. When the second effect (data set 3) and the effect opposite to the first effect (data set 2) (the normalization produced just this effect) are neutralized, we get a normal distribution, with increasing residuals along the *g* score (cf. Murray et al., 2013), and hence the false SLODR effect.

In *data set 5* (real data with the sum score), which contains variables with negative skewness from -.22 to -.36, the results are different for different variables, reflecting their natural differences. Nevertheless, considering them as a whole, one can see residual negative moderation coefficients similar to those of simulation data sets 2 and 3.

Comparing the results obtained in the original real data (sum score) normalization (*data set 6*), we see that the positive residual moderation coefficients for them are less strong than those of data set 4. This shows real data similar to the residual moderation coefficients of data set 2, which are theoretically equal to zero, because normalization restores these data to the simple form of correlated variables without any SLODR effect.

Thus the analysis of data sets 1–6 shows that the real data SLODR-like effect may have been achieved due to similar properties in both cases: the predominance of simpler tasks in the subtests, and the predominance of high-ability persons within the respondents’ distribution (this is only a hypothesis). Actually, only 15% of the tasks were solved by fewer than 40% of respondents, but this fact might be explained by either reason. Such a result coheres with the analysis of the same data that was produced by “traditional methods” (Korneev, Krichevets, & Ushakov, 2019).

*Data set 7* (with the IRT scores) shows the SLODR effect in factor loadings and the opposite tendency in residuals. Such a situation is theoretically possible — the distribution of the results is similar to that of data set 2 — but what it means in terms of intellectual abilities is not a simple question.

## Discussion and Conclusion

We analyzed real data of intellectual tests, choosing four subtests from the total. In all cases, the distribution of results is skewed with a score of about 0.3. The MCFA gave a contradictory result, with negative moderation coefficients for both loadings and residuals. Analysis of the item difficulties shows that the subtests contain more easy items than difficult ones. So the real data result can be explained, at least partly, by irregularity of task difficulty. But the comparison of the real data and data set 2 simulation shows that the loading moderation coefficients of real data are greater modulo than those of data set 2, and, vice versa, the residual moderation coefficients for data set 2 are greater than those of the real data. So there may be some additional effect that can be explained either by SLODR or by case selection.

The similarity of the “true” SLODR model and the normalized case selection model raises the question whether it is possible to differentiate such situations in principle in the classical psychometric framework. In that framework, the normalization is considered as a possible instrument to get an interval scale (Furr & Bachrach, 2008), and just such rescaling converts data set 3 to data set 4, which is very similar to the “true” SLODR, data set 1. We expect that the answer is “no”, but the subject needs detailed exploration.

Not only normalization may lead to this effect. If there is a negatively skewed distribution of participant abilities, we may pick up a set of tasks with a positively skewed distribution of difficulties to make the distribution of sum scores normal, as in the case of data set 4, with the spurious SLODR effect. Note that this effect can be detected by both “traditional” methods (Korneev, Krichevets, & Ushakov, 2019) and the MCFA methods of detection used here, so the skewness itself cannot be completely responsible for the spurious SLODR detection.

At the same time, the IRT approach may reveal both the different densities of easy and difficult tasks and skewed distribution of respondents’ ability, and so can help solve the problem mentioned above.

The two-parameter IRT model method of test scoring could not be considered as fully adequate for our test tasks, due to the presence of a guessing strategy among some participants and its absence among others; but to the extent that it is appropriate, it shows a more complicated situation than the SLODR effect with normal subtest distributions. It shows a decreasing of both intercorrelations and residuals, while ability level is increasing.

An interesting question is to what extent the different easy and difficult task density in the IRT model may lead to spurious detection of a dedifferentiation effect or masking the “true” SLODR effect within the IRT framework (such a hypothesis was formulated by Breit, Brunner, & Preckel, 2020, Study 2), due to different variance of ability estimated at different loci of the ability scale. This may be a subject of future simulation studies.

## Supplementary Materials

The following are available online at http://mathpsy.com/slodr: Table S1: Goodness of fit of the model obtained to different datasets. Appendix 1: Running variation estimate. Appendix 2: SPSS syntax for generation of dataset with SLODR simulation.
